# Loss of murine Paneth cell function alters the immature intestinal microbiome and mimics changes seen in neonatal necrotizing enterocolitis

**DOI:** 10.1371/journal.pone.0204967

**Published:** 2018-10-01

**Authors:** Shiloh R. Lueschow, Jessica Stumphy, Huiyu Gong, Stacy L. Kern, Timothy G. Elgin, Mark A. Underwood, Karen M. Kalanetra, David A. Mills, Melissa H. Wong, David K. Meyerholz, Misty Good, Steven J. McElroy

**Affiliations:** 1 Department of Microbiology and Immunology, University of Iowa, Iowa City, Iowa, United States of America; 2 Stead Family Department of Pediatrics, University of Iowa, Iowa City, Iowa, United States of America; 3 Departments of Pediatrics and Food Science and Technology, University of California Davis, Sacramento, California, United States of America; 4 Department of Cell, Developmental, and Cancer Biology, Oregon Health & Science University, Portland, Oregon, United States of America; 5 Department of Pathology, University of Iowa, Iowa City, Iowa, United States of America; 6 Department of Pediatrics, Washington University School of Medicine, St Louis, Missouri, United States of America; National Cancer Institute, UNITED STATES

## Abstract

Necrotizing enterocolitis (NEC) remains the leading cause of gastrointestinal morbidity and mortality in premature infants. Human and animal studies suggest a role for Paneth cells in NEC pathogenesis. Paneth cells play critical roles in host-microbial interactions and epithelial homeostasis. The ramifications of eliminating Paneth cell function on the immature host-microbial axis remains incomplete. Paneth cell function was depleted in the immature murine intestine using chemical and genetic models, which resulted in intestinal injury consistent with NEC. Paneth cell depletion was confirmed using histology, electron microscopy, flow cytometry, and real time RT-PCR. Cecal samples were analyzed at various time points to determine the effects of Paneth cell depletion with and without *Klebsiella* gavage on the microbiome. Deficient Paneth cell function induced significant compositional changes in the cecal microbiome with a significant increase in *Enterobacteriacae* species. Further, the bloom of *Enterobacteriaceae* species that occurs is phenotypically similar to what is seen in human NEC. This further strengthens our understanding of the importance of Paneth cells to intestinal homeostasis in the immature intestine.

## Introduction

A key regulator of small bowel homeostasis and the intestinal microbiome is the Paneth cell [1]. Paneth cells are granular secretory cells located at the base of the crypts of Lieberkühn. These dense granules contain multiple antimicrobial peptides that are secreted constitutively and in response to bacterial antigens to regulate the intestinal microbiome [2, 3]. The composition of the intestinal microbiota and its interaction with the host tissue is critical in the pathogenesis of many disease processes such as inflammatory bowel disease (IBD) and necrotizing enterocolitis (NEC) [4, 5].

NEC is primarily a disease of premature infants, affecting 4,000 premature infants every year in the US and leading to the death of 1/3 of those infants [6, 7]. The pathophysiology of NEC is postulated to result from bacterial translocation across the immature epithelial barrier, leading to tissue invasion and destruction [8, 9], but the exact mechanisms remain unknown. No single organism has been found to be causative of NEC [10, 11], although multiple studies have associated bacterial dysbiosis and especially a bloom of *Enterobacteriaceae* prior to NEC development [12–15]. This suggests that alterations of the intestinal microbiota are either directly responsible or are an associated marker of NEC development.

Our lab and others have previously shown that infants who developed NEC had significantly fewer Paneth cells than controls [16, 17]. The recent observations that 1) Paneth cell numbers begin to increase in the immature infant small intestine at approximately 29 weeks corrected gestational age [18], 2) *Proteobacteria* are the dominant fecal phylum between 28 and 33 weeks corrected gestational age [13], and 3) the peak incidence of NEC is 28–33 weeks corrected gestational age [19] also suggest a potential role for Paneth cell dysfunction in NEC. As Paneth cells directly affect the composition of intestinal bacteria, it is reasonable to hypothesize that functional depletion of Paneth cells is involved in the dysbiosis observed before or during NEC development. To address this, we utilized chemical and genetic techniques to deplete Paneth cells in the immature intestine and then used *Klebsiella* gavage as our previously described NEC model [20–22] to investigate the role of Paneth cell function on the composition of the microbiome of the immature intestinal tract.

Our initial hypothesis for this study was that Paneth cell depletion would have acute effects on the composition of the immature intestinal microbiome. Our results show that Paneth cell depletion alters the composition of the cecal microbiome acutely and long term after the single initial insult. Furthermore, our data show striking similarities in the composition of intestinal bacteria following Paneth cell depletion-induced NEC to those seen in human infants prior to NEC onset. These results may explain a key mechanism by which the intestinal microbiome is altered prior to development of disease.

## Materials and methods

### Mice

Mice were bred at The University of Iowa under standard conditions according to protocols approved by the Institutional Animal Care and Usage Committee (Approval 7091143). All mice were dam-fed prior to experiments, and unless otherwise indicated, experiments were conducted with postnatal day (P) 14–16 mice. On the day of experimentation, animals were separated from their mothers and maintained in a temperature- and humidity-controlled chamber. All mice were either wild type C57Bl/6J or on a C57Bl/6J background, and founders were purchased from The Jackson Laboratory (Bar Harbor, ME). *PC-DTR* mice were generated by inserting a HA-tagged human diphtheria toxin receptor into the Cryptdin-2 promoter on the surface of Paneth cells. The construct of this vector was a generous gift from Dr. Jeff Gordon at Washington University [23]. *PC-DTR* mice were generated in the University of Iowa Transgenic Mouse Core via pronuclear injection into FVB founders and were crossed to a C57Bl/6J background as previously described [22]. Rosa mice (Gt(ROSA)26Sor^tm4(ACTB-tdTomato,-EGFP)Luo^/J) were purchased from The Jackson Laboratory (Bar Harbor, ME) and were originally on a C57Bl/6J-129 crossed background. To move the strain to a complete C57Bl/6J background, founders were cross bred with Wild type C57Bl/6J animals for 8 generations.

### Bacteria

Unless otherwise noted, all studies were performed using *Klebsiella pneumoniae* 10031 (ATCC, Manassas, VA). Prior to gavage, all bacteria were grown to log-phase and optical density was performed to determine CFU quantity. All mice receiving bacteria were given 1x10^9^ CFUs pathogen/g body weight via single gavage feed. *Klebsiella Zea Mays* is a wild type, GFP labeled *Klebsiella* [24], and was a generous gift from Eric Triplett, University of Florida. To determine bacterial transit time, Rosa mice were gavaged with 1x10^9^ CFU/gbw of GFP-tagged *Klebsiella Zea Mays* and were sacrificed at 30, 60, 90, 120, 180 and 240 minutes post-gavage. The terminal ileum was harvested and examined for the presence of GFP-tagged *Klebsiella*.

Ileal effluents were collected by flushing PBS through the intestinal lumen. Spectrophotometry was used to measure the total bacterial load in the ileal effluent.

### Induction of Paneth cell depletion

#### Dithizone-induced Paneth cell depletion

P14-16 mice were given an intraperitoneal injection with either 33 mg/kg dithizone (Sigma) dissolved in 20% NH_4_OH/EtOH solution, or an equivalent volume of NH_4_HO/EtOH buffer alone [22]. Six hours after injection (time point of greatest Paneth cell reduction[25]), mice were gastrically gavaged with 1×10^9^ CFU bacteria/kg body weight or an equivalent volume of sterile media (nutrient broth; ATCC) [21, 26]. Mice were monitored for 10 hours after gavage and then euthanized for tissue harvesting. Mice were kept separate from their dams during the experiment.

#### Diphtheria toxin-induced Paneth cell depletion

P14-16 *PC-DTR* mice were given an intraperitoneal injection with either 40 ng/g body weight diphtheria toxin (2 μg/μl solution) in phosphate buffered saline (PBS), or an equivalent volume of (PBS) alone [22]. Twenty-four hours after injection, mice were gavaged with 1×10^9^ CFU pathogen/kg body weight or an equivalent volume of sterile media (nutrient broth; ATCC). Mice were monitored for 10 hours after gavage and then euthanized for tissue harvesting. Mice were kept separate from their dams during the experiment.

### Paneth cell quantification

Ileal sections were stained with Alcian Blue/Periodic Acid Schiff stain (Sigma-Aldrich) as previously shown [20]. To minimize sectioning variability, all sections were obtained from the center of the intestinal sample and only areas with full villi were included. In each sample used for measurement, at least 3 distinct areas were counted to minimize sectioning variances. Cells were quantified with a 60x objective (600x total magnification) by a single blinded investigator. Intestinal sections from at least five animals were analyzed for each experimental group and at least 100 crypts were counted per animal. All data were obtained using a Nikon NiU microscope using Nikon Elements software (Nikon). Paneth cell quantification by flow cytometry was performed as described previously [27]. Briefly, ileal samples were removed and flushed with cold PBS, opened lengthwise, and incubated in ice with PBS/30 mM, EDTA/1.5 mM, DTT/10 μm, Y27632 for 20 min prior to transfer to buffer without DTT at 37°C for 10 min. Samples were shaken to dissociate crypts from villi and then centrifuged at 1000 rpm for 5 min. Pelleted cells were washed with PBS, and re-suspended in Hank’s Balanced Salt Solution (HBSS)/0.3 U/ml dispase at 37°C. 150 U/ml DNase I was added and the cellular suspension was passed through 100, 70, and 40 μm cell strainers. Cells were pelleted at 1000 rpm for 5 min, washed with 10 ml 10% FBS, then re-suspended in 3 ml HBSS with 5% FBS. 100 μl of 10 million/ml cells were fixed in 4% paraformaldehyde for 15 min, washed with PBS, and re-suspended in saponin permeabilization buffer with Lyz-fluorescein isothiocyanate antibody (1:10, Dako, Carpinteria, CA) at room temperature for 30 min. All flow analyses were performed using Becton Dickinson LSR II Flow Cytometer (BD Biosciences, CA) and FlowJo software. Nucleated cells were determined through the use of a bivariate side scatter area vs Hoechst 33258 area plot and doublets were excluded by plotting forward scatter width vs PerCP-Cy5.5-A. PerCP-Cy5.5-A was used in place of forward scatter area to allow better separation for distinguishing aggregates using the natural autofluorescence present in the cells. To confirm all doublets had been gated out a bivariate plot of forward scatter area vs forward scatter width was used. FITC-A positive cells could then be distinguished from the non FITC-A expressing cells when plotted vs forwards scatter area.

### Serum collection

Prior to euthanization, blood was obtained from the facial vein as previously described [17]. Whole blood samples were placed on ice for one hour then centrifuged at 7000 RPM for 5 minutes to isolate serum. Cytokines were quantified using a Meso-Scale Discovery V-Plex assay (Meso-scale, Gaithersburg, MD) according to the manufacturer’s instructions. Plates were read on a Sector Imager 2400 at 620 nm.

### Gene expression

For mRNA quantification, ileal samples were homogenized using a TissueLyser LT (Qiagen), as previously described [21, 28, 29]. RNA was isolated using RNeasy Plus Mini Kit (Qiagen) according to manufacturer’s directions. RNA concentration and quality were determined using a NanoDrop 1000 Spectrophotometer (Thermo Fisher Scientific). Quantitative real-time reverse transcription-polymerase chain reaction (qRT-PCR) was performed using Taqman Fast Universal PCR Master Mix (2X) (Life Technologies) and Taqman Gene Expression Assays for cryptdin, and lysozyme, (Life Technologies). qRT-PCR reactions were run in a C1000 Thermal Cycler (Eppendorf) and using the CFX96 Real-Time PCR Detection System (BioRad). Fold change in gene expression was determined by normalizing gene expression to ß-actin in each sample. The 2ΔΔ-CT method was used to compare gene expression levels between samples. RNA-seq reads were aligned to the Ensembl top-level assembly with STAR version 2.0.4b. Gene counts were derived from the number of uniquely aligned unambiguous reads by Subread:featureCount version 1.4.5. Transcript counts were produced by Sailfish version 0.6.3. Sequencing performance was assessed for total number of aligned reads; total number of uniquely aligned reads; genes and transcripts detected; ribosomal fraction known junction saturation and read distribution over known gene models with RSeQC version 2.3. All gene-level and transcript counts were then imported into the R/Bioconductor package EdgeR and TMM normalization size factors were calculated to adjust samples for differences in library size. Ribosomal features as well as any feature not expressed in at least the smallest condition size minus one sample were excluded from further analysis and TMM size factors were recalculated to created effective TMM size factors. The TMM size factors and the matrix of counts were then imported into R/Bioconductor package Limma and weighted likelihoods based on the observed mean-variance relationship of every gene/transcript and sample were then calculated for all samples with the voomWithQualityWeights function. Performance of the samples was assessed with a Spearman correlation matrix and multi-dimensional scaling plots. Gene/transcript performance was assessed with plots of residual standard deviation of every gene to their average log-count with a robustly fitted trend line of the residuals. Generalized linear models were then created to test for gene/transcript level differential expression. Differentially expressed genes and transcripts were then filtered for FDR adjusted p-values less than or equal to 0.05.

### Microbiota analysis

Mice were sacrificed according to institutional guidelines at the University of Iowa. Ceca were removed and placed in 1 mL of *RNA*Later (Sigma Aldrich, St. Louis, MO) and stored overnight at -4°C. The ceca were then transferred to a clean tube and stored at -80°C until processing. Cecal samples were thawed and the ZR Fecal DNA MiniPrep kit (Zymo Research, Irvine, CA) was used to extract DNA from the intact ceca. The extracted DNA was stored at -20°C. Amplification and sequencing were performed as previously described [30, 31]. Bacterial 16s rRNA amplification of the V4 domain was performed using the following primers: F515 (5’-*NNNNNNNN****GT***GTGCCAFCMGCCGCCGCGGTAA-3’) and R806 (5’-GGACTACHVGGGTWTCTAAT-3’), with the forward primer modified to contain a unique 8 nucleotide linker sequence (italicized poly-N section of the primer above) and a 2-nucleotide linker sequence (bold, underlined portion) at the 5’ end. PCR reactions used 5–100 ng DNA template, 1X GoTaq Green Master Mix (Promega, Madison, WI), 1 mmol/L MgCl_2_, and 2 pmol of each primer. PCR was performed at 94 °C for the initial 3 minutes followed by 35 cycles of 94°C for 45 s, 50°C for 60 s, and 72°C for 90 s, with a final extension of 72°C for 10 minutes. PCR amplicons were grouped at approximately equal amplification intensity ratios and were purified using the Qiaquick PCR purification kit (Qiagen). The PCR amplicons were submitted to the UC Davis Genome Center DNA Technologies Core for Illumina paired-end library preparation, cluster generation, and 250 bp paired-end Illumina MiSeq sequencing. Data from the sequencing run was analyzed using the QIIME software package (University of Colorado, Boulder, CO, version 1.9.1) [32]. Sequences were quality filtered and demultiplexed, and the UCLUST (drive5.com, Tiburon, CA) was used to assign operational taxonomic units (OTUs) to the sequences, based on a 97% pairwise identity [33, 34]. Secondary filtration of 0.005% was used to remove low-abundance OTUs [33]. The filtered OTUs were taxonomically classified based on the Ribosomal Database Project classifier (Michigan State University, East Lansing, MI) [35] against a representative subset of the Greengenes 16s rRNA database (Second Genome, South San Francisco, CA, gg_13_5 release) [36]. OTU sequence alignment was performed using PyNAST (University of Colorado) [33, 37] and was used to construct a phylogenetic tree for β diversity analyses. β diversity was estimated by calculating unweighted and abundance-weighted UniFrac distances [38]. Sample clustering was based on between-sample distances.

### Microscopic examination

Samples were deparaffinized and rehydrated. To unmask antigens, citrate buffer (pH 6.0) was used in a Biocare Company Decloaking Unit at 110 degrees for 15 minutes followed by TBST washing (5 minutes x 2) and blocking in 5% normal goat serum (Cell Signaling). Rabbit-Anti HA (Abcam, Cambridge, MA), chicken-anti GFP (Aves Laboratories, Tigard, OR), anti-lysozyme (Invitrogen, Waltham, MA) were used as primary antibodies at manufacturers recommended concentrations. D1C2 antibody was developed through the Intestinal Stem Cell Consortium (https://iscconsortium.org/). Sections were incubated with goat anti-rabbit Alexa Fluor 488 at 1:2000 or goat anti-chicken Alexa Fluor 488 1:1000 (Invitrogen, Waltham, MA) for 45 minutes at room temperature, washed 5 minutes x3 with PBS, and slides were mounted with hard set fluorescence mounting medium (Vector Laboratories, Burlingame, CA). Images were captured using confocal microscopy. For ultrastructural examination, 1–1.5 mm sections of distal ileum ring fragments were obtained from all groups and fixed in 2.5% glutaraldehyde (in 0.1 M sodium cocadylate buffer [pH 7.4]) overnight at 4°C. Following fixation, samples were post-fixed with 1% osmium tetroxide for 1.5 h and then dehydration with ethanol and embedded in Epon 12 (Ted Pella, Redding, CA). Ultrathin sections (70 nm) were post-stained with uranyl acetate and lead citrate and viewed with a JEOL 1230 transmission electron microscope (TEM) (Tokyo, Japan).

### Statistical analysis

All experiments were performed in at least triplicate and all experiments had an n of 3–10 animals. Specific sample sizes are denoted in the Results. ANOVA and non-parametric Kruskal-Wallis testing was performed to determine statistical significance using Graph Pad Prism v6. Significance was set as P < 0.05 for all experiments.

## Results

### Dithizone significantly disrupts Paneth cell number and function in C57Bl6 mice

**Fig 1:**
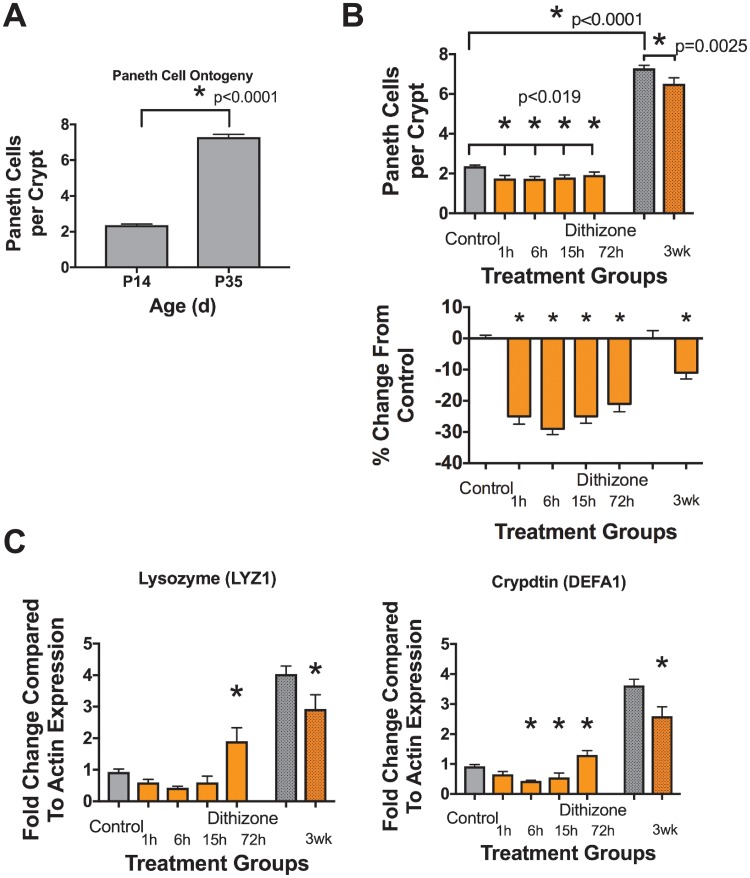
Dithizone significantly depletes Paneth cells in immature intestine. Paneth cells were quantified per crypt in normal tissue. (A) Mice at age P35 have significantly more Paneth cells than at P14 (n = 6 for all treatment groups, p < 0.0001). (B) Dithizone exposure significantly reduces Paneth cell counts acutely (20–30% reduction) and chronically (11%) compared to age matched controls (n = 6 for all treatment groups, p < 0.019). (C) In addition to reduction in cell counts, dithizone exposure also induces significant decreases in Paneth cell-specific genes (n = 5 for all treatment groups, p < 0.019 for all significant points).

Baseline quantification of Paneth cells numbers in C57Bl6 mice from P14 and P35 were obtained by staining ileal sections with Alcian Blue-Periodic Acid Schiff stain and positive cells were counted per crypt as previously described [20, 22, 29]. In P14 animals, there were 2.4 ± 0.3 Paneth cells per crypt. This ratio significantly increased over time to 7.3 ± 0.4 Paneth cells per crypt at P35 (Fig 1A, n = 6 for all treatment groups, p < 0.0001). To quantify the effect of dithizone-induced depletion on Paneth cells, P14 mice were treated with dithizone and euthanized at 1, 6, 15, and 72 hours following treatment (Fig 1B). Paneth cell numbers significantly decreased 1 hour after dithizone administration compared to controls (2.4 ± 0.3 vs 1.8 ± 0.3, n = 6 for all treatment groups, p = 0.004). Paneth cell counts remained significantly less, but gradually increased over 72 hours following exposure (Fig 1B, 2.4 ± 0.3 vs 1.7 ± 0.2 at 6 hours, 1.8 ± 0.3 p = 0.005 at 15 hours and 1.9 ± 0.3, p = 0.02 at 72 hours respectively, n = 6 for all treatment groups, p < 0.019 for all points). To quantify any chronic effects of dithizone-induced Paneth cell depletion, mice were treated with dithizone at P14 and returned to their mothers (Fig 1B). Three weeks after treatment (P35), the mice were euthanized and their ilea were harvested as above. At three weeks following dithizone administration, Paneth cell counts remained significantly decreased compared to control mice (Fig 1B, 7.3 ± 0.4 vs 6.5 ± 0.7, n = 6 for all treatment groups, p = 0.004). To further measure depletion of the Paneth cell function, tissue mRNA expression of the antimicrobial peptides lysozyme and cryptdin was quantified by real time rtPCR at the same time points as above. Both lysozyme and cryptdin were significantly decreased by 1 hour following dithizone exposure and remained significantly below control levels (Fig 1C, n = 5 for all treatment groups, p < 0.019 for all significant points).

### Dithizone and DTX induced Paneth cell depletion induces changes to the composition of the cecal microbiome

**Fig 2:**
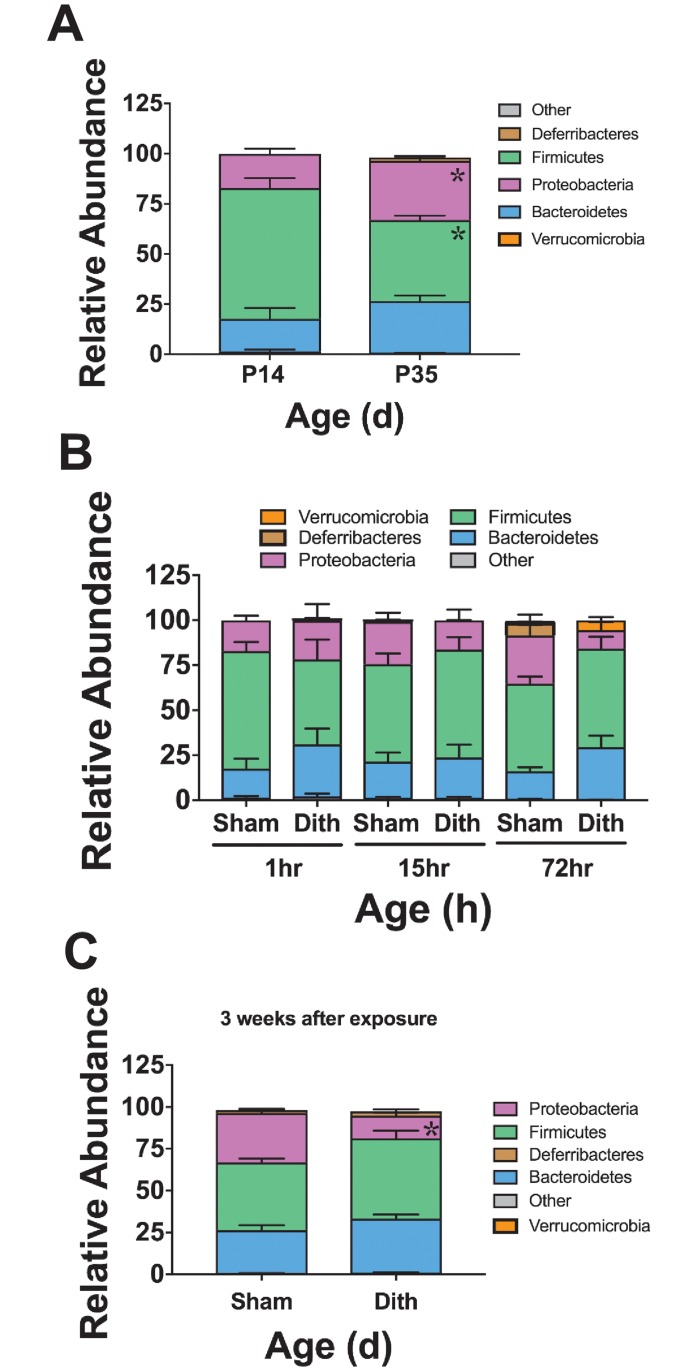
Dithizone induced-Paneth cell depletion causes initial non-significant disturbances to the cecal microbiome composition that develop into lasting significant alterations. (A) The cecal microbial population significantly changes during normal aging in C57BL6 mice (n = 6 animals in each group, p < 0.006 for all significant points). (B) Dithizone-treated mice exhibited increased relative percentages of *Bacteroidetes* along with decreased relative percentages of *Firmicutes* and *Proteobacteria* compared to the intestinal microbiota of controls; however, none of the alterations reached statistical significance. (C) However, these non-significant population shifts in *Bacteroidetes* (26% sham vs 32% dithizone) and *Firmicutes* (40% vs 48%) persist up to 3 weeks following Paneth cell disruption, and the alterations in *Proteobacteria* become significantly different from sham controls (30% vs 14%, p < 0.0001). (n = 6 for all treatment groups).

Examination of the baseline cecal bacterial composition in P14 C57Bl6 mice revealed a biome composed mainly of organisms from the Phyla *Bacteroidetes*, *Proteobacteria*, and *Firmicutes* (Fig 2A). Compared to mice at age P14, mice at age P35 had significant increases in the number of *Proteobacteria* (p < 0.015) and *Firmicutes* (p < 0.0001) (n = 6 animals in each group, Fig 2A). To determine the acute effect of dithizone-induced Paneth cell depletion on the bacterial composition of the ileum, cecal samples were obtained from 6 mice at 1, 15, and 72 hours after dithizone exposure and 6 mice at equivalent time points receiving sham treatment. Paneth cell depletion induced bacterial compositional shifts but was not associated with any statistically significant alterations in the microbial community between 1 and 72 hours following exposure (Fig 2B).

To quantify any chronic effects of dithizone-induced Paneth cell depletion, mice were treated with dithizone at P14 and returned to their mothers in the same small animal housing room. Three weeks after treatment (P35), the mice were euthanized and their cecal samples were collected and examined as above. Microbiome alterations following dithizone-induced Paneth cell depletion, although not statistically significant in the short term, exhibit significant changes at three weeks. *Proteobacteria* composed the most significant change in microbial community composition three weeks after treatment as the phylum suffered a decline (30% vs 14%, p < 0.0001), compared to control mice of the same age (n = 6 for all treatment groups, Fig 2C).

**Fig 3:**
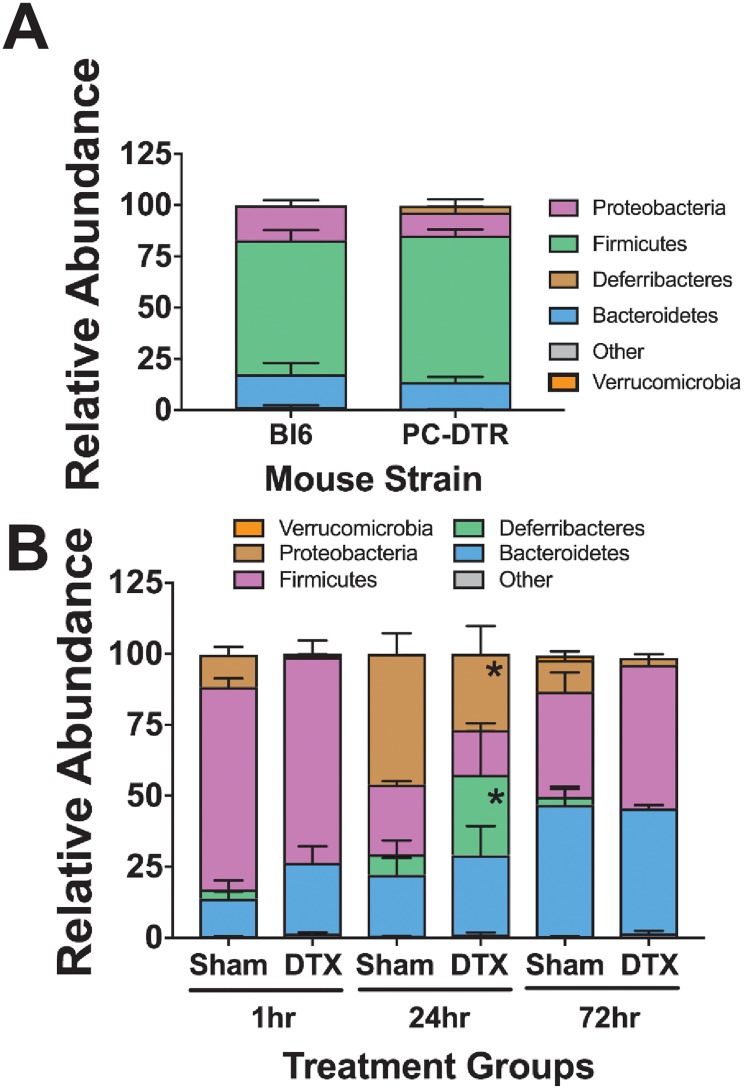
Paneth cell-depletion induced changes in the microbiome are not dependent on dithizone. Normal cecal microbiomes were compared between P14 non-treated wild type C57Bl6 mice and *PC-DTR* mice (on C57Bl6 background). (A) *PC-DTR* mice had no significant differences in taxa compared to wild type mice of the same age n = 6 for each treatment. (B) Paneth cell depletion using DTX induces significant changes in the microbiota composition by 24 hours in *Deferribacteres* (7% in Sham vs 28% in DTX treated, p = 0.0004) and in *Proteobacteria* (46% in Sham vs 27% in DTX treated, p = 0.001), but these significant changes disappear and shift back towards normal floral composition after 72 hours.

To determine if our findings were dependent on the type of Paneth cell depletion, we examined changes in cecal microbiome composition using our previously described mouse line (*PC-DTR*) which has a human diphtheria toxin receptor bound to the Cryptdin-2 promotor on the Paneth cell membrane [22]. Treatment of this mouse with diphtheria toxin (DTX) results in Paneth cell-specific loss [22]. The cecal microbiota at P14 was compared between control C57Bl6 mice and control *PC-DTR* mice to evaluate for strain differences. The predominant organisms in the *PC-DTR* mice were statistically equivalent to those in C57Bl6 mice and included *Firmicutes*, *Bacteroidetes*, and *Proteobacteria*. To determine the effects of Paneth cell depletion on the microbial community of *PC-DTR* mice, cecal samples were collected 1, 24 and 72 hours after DTX exposure. Paneth cell depletion from DTX exposure in *PC-DTR* mice resulted in significant acute alterations to the microbial composition, which trend back towards normal over time. The most significant change was seen in the 24 hour post-treatment time point although minor differences were also seen at 1 hr and 72 hrs post treatment. Importantly, the two models of Paneth cell depletion, dithizone and DTX, induce distinct changes to the composition of the cecal microbiome (n = 6 for all treatment groups at all time points, Figs 2B and 3C).

### Dithizone and DTX induce Paneth cell depletion through different mechanisms

**Fig 4:**
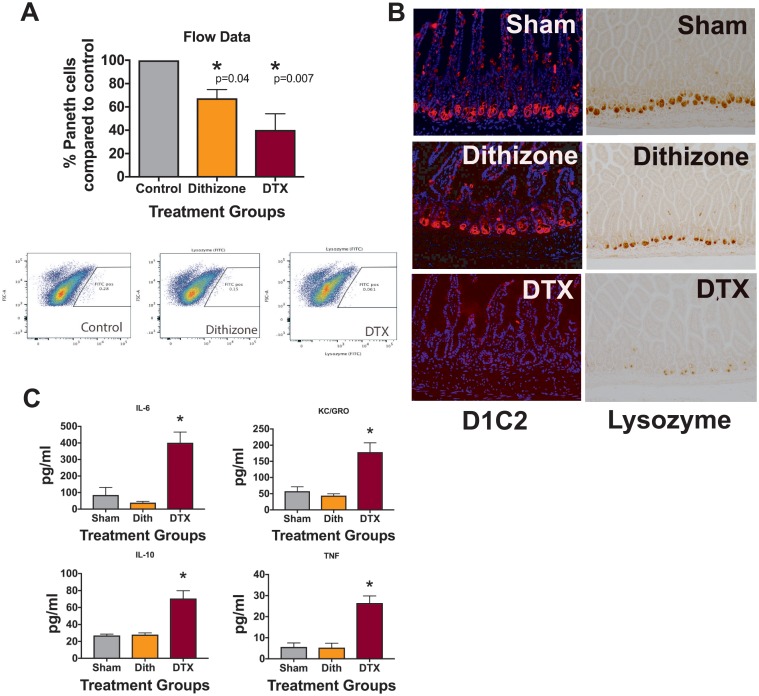
Dithizone and DTX work through different mechanisms to disrupt Paneth cells. (A) Dithizone treatment reduces lysozyme containing Paneth cells by 33% while DTX treatment reduces Paneth cells by 60% using flow cytometry for anti-lysozyme (n = 3 per group). (B) Examination of Paneth cell histology by lysozyme following treatment with dithizone or DTX shows a loss of lysozyme stained cells compared to controls. However, examination of Paneth cell histology using the novel D1C2 antibody that appears to target Paneth cells shows a loss of cells following DTX, but not following dithizone (n = 3 per group, representative samples shown). (C) Serum levels of IL-10, IL-6, TNF and KC-GRO 16 hours following treatment with DTX show significantly increased levels compared to sham controls (n = 5, p < 0.0069 for all cytokines) while dithizone does not cause any significant changes.

Since our complementary Paneth cell depletion models produced different phenotypes in the cecal microbiome, we wanted to further examine potential mechanistic causes for these differences. To rigorously evaluate our quantification of Paneth cell loss, we examined Paneth cell counts using flow cytometry for lysozyme. Similar to our data using immunohistochemistry, this methodology showed that Dithizone reduced lysozyme containing Paneth cells by 33% while DTX reduced them by 60% (n = 3, p = 0.4 for dithizone and 0.007 for DTX, Fig 4A). One difficulty of Paneth cell biology is that there are currently no cell surface markers commercially available. However, the Wong lab has recently developed a novel antibody (D1C2) that recognizes the cell surface of Paneth cells and was generated as an Intestinal Stem Cell Consortium funded project (https://iscconsortium.org/). Using this reagent, we examined the effect on crypts of animals treated with dithizone or DTX compared to sham controls (Fig 4B). As predicted, DTX eliminated both lysozyme and D1C2 staining in the small intestinal crypts. However, while dithizone treatment reduced lysozyme staining, it had no effect on D1C2 staining patterns. As DTX only affects cells that contain a human diphtheria toxin receptor (only Paneth cells in the PC-DTR mice), this suggests that while DTX induces cellular necrosis [23], dithizone may induce Paneth cell dysfunction through an alternative mechanism. Examination of serum cytokines also supports a different mechanism (Fig 4C). DTX treatment significantly increases serum Interleukin (IL) 6 and10, as well as, KC-GRO (murine equivalent of IL-8), and tumor necrosis factor (TNF) compared to sham controls (n = 5 for all groups, p < 0.007 for all groups). Dithizone, on the other hand, had no significant effect on serum cytokine levels.

**Fig 5:**
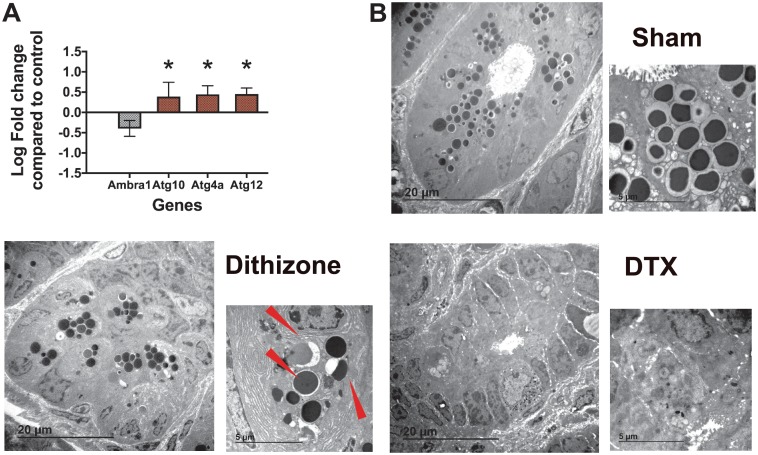
Dithizone induces autophagy-like changes in Paneth cells. (A) Treatment with dithizone induces a significant increase in *Atg10* (p = 0.03), *Atg4a* (p = 0.0004), and *Atg12* (p = 0.000006), as well as a significant decrease in the beclin1 regulator *Ambra1* (p = 0.0003). (n = 5 for each group). (B) Cellular examination using transmission electron microscopy shows presence of autophagosomes (red arrows) in animals treated with dithizone, but not in those treated with DTX or in sham controls. (n = 3 per group). EM sections were evaluated by a single blinded investigator.

Recent studies have elucidated secretory autophagy as a mechanism of Paneth cell granule release [39–41]. Mice lacking a normal autophagy pathway have decreased Paneth cell lysozyme granule secretion and defects in bacterial clearance [42]. To see if dithizone played a role in autophagy genes, small intestinal samples were examined by RNAseq for dithizone-induced changes (Fig 5A). Dithizone treatment induces significant changes in several autophagy genes, including increases in *Atg10*, *Atg4a*, and *Atg12*, as well as decreasing the beclin1 regulator *Ambra1*. Because autophagy is a complex system, we next examined our tissue samples under electron microscopy. While samples treated with DTX showed signs of necrosis such as cytoplasm disorganization and disruption of plasma and nuclear membranes, samples treated with dithizone showed characteristics of autophagy including autophagosomes (Fig 5B). This further suggests that the mechanisms of Paneth cell depletion in dithizone and DTX treatments are dissimilar.

### Both methods of Paneth cell depletion-induced NEC result in an *Enterobacteriaceae* bloom

**Fig 6:**
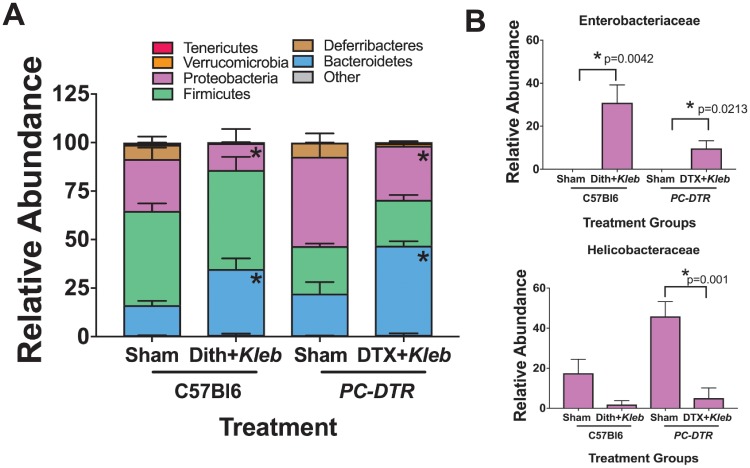
Paneth cell depletion-induced NEC results in *Enterobacteriaceae* blooms similar to patterns seen in human patients. (A) Paneth cell depletion followed by Klebsiella exposure induces significant alterations to the cecal bacterial population in both dithizone and DTX models (n = 6 for all treatments at all time points, all significant p values are < 0.05). (B) Furthermore, Paneth cell depletion induced NEC induces significant blooms of *Enterobacteriaceae* which has been reported in human disease. This bloom corresponds with a significant decrease in *Helicobacteraceae*.

Our laboratory has previously shown that Paneth cell depletion followed by enteral exposure to *Klebsiella* induces NEC-like small intestinal injury [22]; however, these experiments did not evaluate the effect of injury induction on the microbiome. In the dithizone-*Klebsiella* model and in the *PC-DTR* mice exposed to DTX and *Klebsiella*, there was a significant increase in the number of *Bacteroidetes* and a significant compensatory decrease in the relative percentage of *Proteobacteria* (Fig 6A). Interestingly, both methods induced a significant bloom of *Enterobacteriaceae* despite an overall decrease in *Proteobacteria* in the NEC models at the Phyla level (Fig 6B). This bloom was not seen in the control mice or the mice exposed to Paneth cell depletion without bacterial gavage in either the dithizone or the DTX model. The *Enterobacteriaceae* bloom was matched by a significant decrease in *Helicobacteraceae*, also part of the *Proteobacteria* phylum.

### The *Enterobacteriaceae* bloom seen following Paneth cell depletion-induced NEC is independent of gavaged *Klebsiella pneumonia*

**Fig 7:**
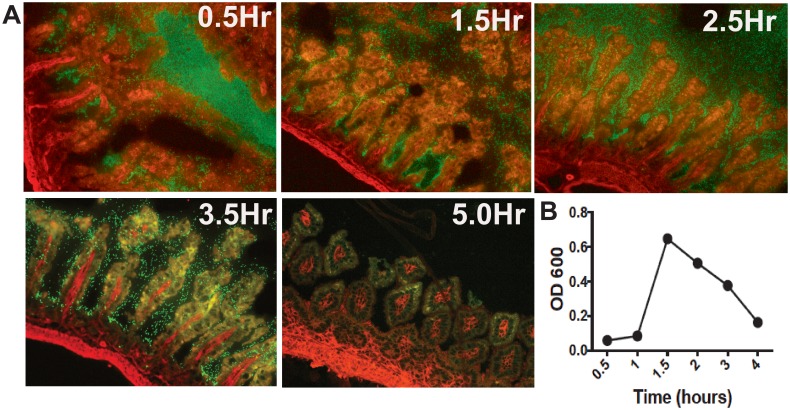
*Enterobacteriaceae* blooms seen in Paneth cell depletion-induced NEC are independent of gavaged bacteria. To determine if the bloom of *Enterobacteriaceae* was due to the Klebsiella gavage, tomato mice with constitutively expressed red fluorescent protein were gavaged with GFP labeled *Klebsiella*. Small intestinal samples were harvested and examined for presence of GFP-tagged *Klebsiella*. (A) As seen above, *Klebsiella* was gone from the small intestine by 5 hours, well before the bloom was seen in dithizone or DTX models (n = 3 animals at each time point, representative microscopy shown). (B) To determine bacterial load, cecal samples were measured by spectrophotometry. Bacterial levels returned to pre-gavage levels within 4 hours of gavage (n = 3).

Since *Klebsiella* belongs to the *Enterobacteriaceae* family (*Proteobacteria* phylum), we lastly wanted to determine if the bloom of *Enterobacteriaceae* seen in our model was simply a result of the gavaged bacteria in our NEC model. Timed samples from Rosa mice gavaged with GFP-labeled Klebsiella were harvested and evaluated. Tagged *Klebsiella* were present in the ileum as early as 30 minutes after gavage and absent from the small intestine by 5 hours (Fig 7A). Additionally, spectrophotometry was used to measure the bacterial load present in the ileal effluent (Fig 7B). Overall, bacterial content in the small intestine increased sharply between 1 and 1.5 hours after *Klebsiella* gavage, and then gradually returned to baseline levels within 4 hours after bacteria administration. The bloom of *Enterobacteriaceae* present following induction of clinical NEC via Paneth cell depletion was seen more than 8 hours after *Klebsiella* gavage, well after the administered *Klebsiella* was shown to have passed through the small intestine.

## Discussion

The host-bacterial axis that exists between humans and their intestinal microbes is critical to maintenance of health. Neonates are exposed to an environment teeming with bacteria at birth leading to sequential colonization by waves of bacteria in predictable patterns [13, 43]. This bacterial maturation is influenced by many factors including, gestation at birth, antibiotic exposure, and diet [4, 30]. A key regulator of homeostasis is the Paneth cell [1]. Loss of Paneth cells and alterations of the intestinal microbiome have both been associated with development of NEC. However, it is unclear if depletion of Paneth cells is causative of the dysbiosis seen prior to NEC or merely an associated finding. Our data clearly show that Paneth cell depletion induces time-dependent changes in the microbiota of the immature small intestine. Alterations in the relative percentages of different phyla occur as early as 60 minutes after Paneth cell depletion, and significant alterations of the biome persist up to three weeks after Paneth cell depletion compared to age-matched controls. Importantly, we also show depletion of Paneth cells followed by exposure to *Klebsiella* not only induces intestinal injury that resembles human NEC [22], but also induces alterations in the intestinal microbiome that mimic what is seen in human infants who develop NEC particularly when looking at the *Enterobacteriaceae* family [14]. This further strengthens the link between Paneth cell depletion and development of NEC-like pathophysiology.

Paneth cells represent an important component of our intestinal innate immunity, preventing translocation and overgrowth of potential pathogenic bacteria [44]. Constitutive and acute secretion of antimicrobial peptides contained in Paneth cell granules helps to keep the small intestinal crypts semi-sterile and modulate the intestinal microbiome [2]. However, preterm infants have underdeveloped Paneth cells and an abnormal microbiome [13, 18]. Our lab has shown that Paneth cells in the immature intestine can be depleted by many factors including inflammation [29] and intrauterine growth restriction [45]. These mechanisms may further decrease an already underdeveloped innate immune system. Thus, understanding the role that Paneth cells play in host physiology in the immature intestine is critical for physicians to properly manage premature infants.

The findings from the above studies support the idea that Paneth cell physiology is important to the host/microbiome axis in the immature intestine. Our data show that depletion of Paneth cells causes alteration in the microbiome composition acutely in the 72 hours following exposure and continues through the next several weeks of life despite no new interventions. It is also interesting to note that our two complimentary models of Paneth cell depletion using dithizone or DTX, induce significant but different changes in the microbiome (Figs 2 and 3). While initial exposure to dithizone expands the predominance of *Bacteroidetes* and diminishes the relative amount of *Firmicutes*, treatment with DTX induces an expansion of *Firmicutes* and a decrease in *Proteobacteria*. In considering why these two treatments induce different compositional changes in the biome, we first considered their mechanisms of action. Dithizone is a heavy metal chelator and acts on Paneth cells by binding to their abundant zinc stores, which potentially deprives bacteria of this essential micronutrient [25, 46]. However, our previous work has shown that dithizone treatment does not induce significant differences in host serum zinc levels [22]. Although DTX clearly induces cellular necrosis, the mechanism of dithizone is less clear. The mechanism by which dithizone facilitates gut injury has been argued to be via cellular necrosis [25, 46]. Our data contradict this notion and alternatively suggest dithizone does not induce Paneth cell necrosis, but perhaps utilizes autophagy pathways. This is an interesting finding as disrupted Paneth cell secretion through altered autophagy pathways has been suggested to be involved in some forms of Crohn’s disease [39, 42]. While further mechanistic data is outside the scope of this manuscript, this gap in knowledge should be further examined.

Importantly, our data show that both models of Paneth cell depletion induced NEC show significant blooms of the *Enterobacteriaceae* family, part of the *Proteobacteria* phylum regardless of the fact that in both scenarios the proportion of *Proteobacteria* decreased. This bloom was not seen in any of the control mice or in the dithizone-only or DTX-only cohorts. Multiple human NEC studies have demonstrated increased numbers of *Enterobacteriaceae* prior to development of NEC [12–15]. Thus, our model of Paneth cell depletion followed by bacterial dysbiosis not only induces a phenotype that is similar to what is seen in human NEC [22], but also induces alterations in the intestinal microbial community that are similar to those seen prior to the onset of NEC. Differences in the overall composition of the microbiome may be due to the difference in the magnitude of inflammation induced (Fig 4C).

Recognizing that administration of *Klebsiella*, a genus in the *Enterobacteriaceae* family, to the treatment mice could potentially confound our results, we performed additional studies to determine if the *Enterobacteriaceae* bloom included the gavaged *Klebsiella*. Overall, bacterial load, determined by spectrophotometry, showed that small intestine bacteria levels rose dramatically between 1 and 1.5 hours following *Klebsiella* gavage. Bacterial levels returned to baseline within 4 hours after gavage. Ileal samples from Rosa mice gavaged with GFP-labeled *Klebsiella* demonstrated that GFP-labeled *Klebsiella* was no longer present in the small intestine by 5 hours post-gavage. These results confirm that the administered *Klebsiella pneumonia* exited the small intestine before the bloom of *Enterobacteriaceae* was seen in the dithizone and DTX NEC models.

In summary, our data clearly show that Paneth cell depletion in the immature small intestine induces significant alterations of the composition of the intestinal microbiota. Our data also show that Paneth cell depletion followed by *Klebsiella*-induced dysbiosis in mice induces a similar phenotype to what is seen in the microbiota of human infants who develop NEC. These results provide support to the idea that Paneth cell depletion or dysfunction may play a key role in development of intestinal disease, especially NEC. However, further investigation is needed to determine if dysbiosis is a causative factor or a result of progression to NEC-like injury.
